# Availability and acceptability of HIV counselling and testing services. A qualitative study comparing clients’ experiences of accessing HIV testing at public sector primary health care facilities or non-governmental mobile services in Cape Town, South Africa

**DOI:** 10.1186/s12889-015-2173-8

**Published:** 2015-09-02

**Authors:** Sue-Ann Meehan, Natalie Leon, Pren Naidoo, Karen Jennings, Ronelle Burger, Nulda Beyers

**Affiliations:** Desmond Tutu TB Centre, Department of Paediatrics and Child Health, Faculty of Medicine and Health Sciences, Stellenbosch University, Francie van Zijl Ave, Parow, Cape Town, South Africa; Health Research Unit, South African Medical Research Council, Francie van Zijl Ave, Parow, Cape Town, South Africa; City of Cape Town Health Directorate, Cape Town, South Africa; Department of Economics, Stellenbosch University, Stellenbosch, Cape Town South Africa

## Abstract

**Background:**

The South African government is striving for universal access to HIV counselling and testing (HCT), a fundamental component of HIV care and prevention. In the Cape Town district, Western Cape Province of South Africa, HCT is provided free of charge at publically funded primary health care (PHC) facilities and through non-governmental organizations (NGOs). This study investigated the availability and accessibility of HCT services; comparing health seeking behaviour and client experiences of HCT across public PHC facilities (fixed sites) and NGO mobile services.

**Methods:**

This qualitative study used semi-structured interviews. Systematic sampling was used to select 16 participants who accessed HCT in either a PHC facility (8) or a NGO mobile service (8). Interviews, conducted between March and June 2011, were digitally recorded, transcribed and where required, translated into English. Constant comparative and thematic analysis was used to identify common and divergent responses and themes in relation to the key questions (reasons for testing, choice of service provider and experience of HCT).

**Results:**

The sample consisted of 12 females and 4 males with an age range of 19–60 years (median age 28 years). Motivations for accessing health facilities and NGO services were similar; opportunity to test, being affected by HIV and a perceived personal risk for contracting HIV. Participants chose a particular service provider based on accessibility, familiarity with and acceptability of that service. Experiences of both services were largely positive, though instances of poor staff attitude and long waiting times were reported at PHC facilities. Those attending NGO services reported shorter waiting times and overall positive testing experiences. Concerns about lack of adequate privacy and associated stigma were expressed about both services.

**Conclusions:**

Realised access to HCT is dependent on availability and acceptability of HCT services. Those who utilised either a NGO mobile service or a public PHC facility perceived both service types as available and acceptable. Mobile NGO services provided an accessible opportunity for those who would otherwise not have tested at that time. Policy makers should consider the perceptions and experiences of those accessing HCT services when increasing access to HCT.

## Background

In South Africa, 12 % of the population (6.4 million) are living with HIV [1]. The AIDS epidemic is generalised with primarily heterosexual transmission [2]. Overall HIV prevalence is highest among females (14 %) compared to males (10 %) and among urban informal dwellers (20 %) compared to those living in rural informal areas (13 %) [1]. More than half (56 %) of the burden lies within the poorest 40 % of the population [3]. Around Cape Town the antenatal prevalence ranges between 9 and 37 % across health sub-districts [4]. Access to HIV services needs to be in line with demographic and socioeconomic factors.

HIV counselling and testing (HCT) is a fundamental component of HIV care and prevention. In 2012, 65 % of South Africans reported to have ever tested for HIV of which 66 % tested in the previous 12 months [1]. Although indicative of fairly good testing coverage, it falls short of the Government’s call for all to test annually [5] and many individuals, including those most likely to be living with HIV, still do not know their HIV status [6]. Public health facilities cannot test everyone as some populations for example males do not readily access public health facilities [7]. In striving toward universal access to HCT, a better understanding of the factors that enable and constrain access to HCT is important and diverse testing opportunities should be considered in an attempt to increase access to HCT.

“Access” is an important concept in health services research [8]. However, it is a multidimensional and complex term [9] that has not been precisely defined [10] nor measured in a consistent manner [9]. Although it is widely accepted that access to health services, refers to the opportunity to use a health service [3], utilization of health services is only one indicator of access having been realized [11] and is insufficient on its own to determine access. A broader interpretation includes utilization of a health service being dependent on three dimensions of access that are conceptually clear and separate [12]; availability, acceptability and affordability [13, 14]. Within this framework availability is concerned with health services being supplied in the right place and time to meet the needs of the population [12], acceptability is concerned with the provider and patient attitudes and expectations of each other and affordability measures the relationship between the cost to the patient to use the service and their ability to pay [14].

This study focuses on the dimensions of availability and acceptability and investigated reasons why clients choose to have an HIV test, why they chose either a public funded primary health care facility or a non-governmental mobile facility as service provider and compared their experiences of HCT. As HCT is offered free of charge at both service providers, affordability was excluded from this study. This study therefore compares participants who accessed either one of two HCT service types.

## Methods

### Setting

In the Cape Town district of the Western Cape Province of South Africa, HCT is routinely provided at publically funded primary health care facilities, district hospitals, at secondary and tertiary level care. There are approximately 142 public primary health care (PHC) facilities offering free HCT services. HCT services are also provided free of charge through non-governmental organizations (NGO).

Since 2008, the Desmond Tutu TB Centre (DTTC) at Stellenbosch University has worked in partnership with non-governmental organizations to establish Community HCT centres in 8 communities around the Cape Town metropole in the Western Cape Province of South Africa. These services are provided either from community-based centers or on a mobile basis, whereby non-permanent structures are set up in appropriate spaces within communities to specifically target groups that do not typically access facility-based services [15].

Stellenbosch University provides overall management, technical assistance and quality assurance and contracts local NGOs to provide HCT services from either the community HCT centres or on an outreach, mobile basis. Mobile services are provided from pop-up tents and a caravan (mobile van) set up in an appropriate open space, including public transport hubs such as taxi ranks, train stations or open fields.

This study was embedded within a larger study, which compared the characteristics of clients who accessed HIV testing across public PHC facilities and NGO mobile services [16]. This larger study took place in the above-mentioned eight communities on the outskirts of the city of Cape Town. These communities are under-developed, densely populated, have a mixture of formal and informal dwellings and are characterised by high unemployment, socio-economic challenges and a high HIV and TB burden.

The larger study mapped a geographical area within each of these 8 communities, by plotting a 2 km radius around each of eight pre-existing NGO community-based HCT centres. All public PHC facilities that fell within this 2 km radius were included in the study. In each of the 8 areas, between one and four public PHC facilities fell within this 2 km radius. There was no overlap between areas. The study focused on comparing the HCT services of mobile funded NGO providers and public PHC facilities within these areas. Services were comparable in terms of operating hours and neither provider type charged a fee.

### Design and sampling

This is a descriptive qualitative study. For the purposes of this study, two of the eight geographical areas were purposively sampled to ensure the inclusion of an ethnically diverse set of respondents. In area one, 1 NGO mobile service and two public PHC facilities were included and in area two, 1 NGO mobile service and 4 public PHC facilities were included.

This study was designed to specifically compare issues around availability and acceptability of HCT services at two existing HCT service types, as reported by those who accessed HCT. Therefore sampling included only those who accessed HCT at either of these two service types.

A total of sixteen adult participants (>18 years) were enrolled in the study. Lists containing the participant’s barcoded study identity number from the larger study were used to systematically sample every tenth participant from those who had attended the mobile services and each PHC facility within the selected areas until 8 participants were enrolled from each service type. In area one, 4 participants were sampled from the mobile and two from each of the two PHC facilities. In area two, 4 participants were sampled from the mobile and one from each of the four PHC facilities. Including participants from different facilities allowed for client experience across several health facilities to be explored.

Each sampled individual was contacted telephonically and a date set for the interview. If the individual was not able to be contacted or declined to be interviewed, the next individual on the list was contacted until for each area, the required number of participants gave permission to be interviewed. In order to obtain permission from 8 participants who had attended the NGO mobile services, 24 were sampled from the lists of which 15 were not contactable (no answer, had moved or had missing contact details) and 1 declined. In order to obtain permission from 8 participants who had attended the public PHC facilities, 19 were sampled: 10 were not contactable and 1declined.

### Data collection

Semi-structured interviews were conducted between March and June 2011, approximately 1 month after the participants’ HIV testing experience. One male and two female interviewers, with experience in qualitative interviewing and from similar ethnic groups to the participants did the interviews in the preferred language of the participant (Afrikaans, isiXhosa or English). To minimise potential bias during data collection the interviewers were not part of the larger initial study and were not aware of the participants’ HIV status.

All interviewers were trained on the overall objective of the study and in using the interview guide as a tool. This guide comprised 3 global topics; participant demographics, health seeking behaviour and HCT experience. The semi-structured interview tool was developed in English and was not translated into any other language. The interviewers could speak and understand English well. The training they received allowed them to translate any questions if required as they conducted the interviews. Interviews were conducted in the communities, predominantly in participants’ homes or a private space elsewhere, including in the interviewer’s car. The decision regarding where to hold the interview was a mutual one between interviewer and participant based on safety and privacy. Interviews varied between twenty and sixty minutes, were digitally recorded and transcribed by each interviewer. Interviews conducted in a language other than English were translated during transcription.

### Analysis

Each interview transcription was read and reread by SM, to ensure familiarity with the content. A constant comparative analysis was done to identify common and divergent responses in relation to the key questions and categorised and abstracted to form common themes. A sample of four transcripts (two from PHC facilities and two from mobile services) were independently analysed by a co-author (NL). There were no discrepancies in the analysis between the two researchers. Those involved in data analysis were blinded to participant HIV status.

Two identical matrixes, one each for NGO mobile and public PHC facilities, were developed in Microsoft Excel 2010. These were used to categorise and compare data across provider types. Health seeking behaviour and HCT experience were entered as pre-determined global themes in the first column. The main interview questions related to each of these global themes. Participant responses to these global themes were organised around sub-categories, which were entered underneath the relevant global theme. These sub-categories were aspects of the global themes that the researchers were interested in and formed minor questions (prompts) on the interview schedule, meaning that they were only used if the participant provided insufficient detail when responding to the main interview questions. Within each of these sub-categories, responses were compared across participants and themes emerged within each sub-category. The matrix is illustrated in Table 1. A general comments column was included to note consistent and divergent responses across HCT provider types.

**Table 1:** Illustration of the data analysis matrix

Global theme: Health seeking behaviour				
Sub-category	Emergent themes	Participant 1 barcode	Participant 2 barcode	Participant 3 barcode
Reason for testing	Opportunity	“…on the way we saw the tents on the side of the road with a sign that says HCT”.		
	Affected by HIV	“I have a sister in XXX who is HIV positive…”		“have a cousin who is HIV positive…she died in January..”
	Perceived personal risk for HIV		“… I had a boyfriend whom I did not trust, and then I thought for my sake I needed to know my status”.	“My boyfriend doesn’t want to use a condom…”

### Ethics approval

The study was approved by the Health Research Ethics Committee of Stellenbosch University (N10/09/288) and The International Union against Tuberculosis and Lung Disease Ethics Advisory Group (EAG 58/10). All participants were part of the larger study and had already provided written consent, which included the possibility of being contacted for face to face interviews. When contacted telephonically, participants could decline to be interviewed and those interviewed were free to end the interview at any point. Participants were not given any incentives for taking part.

## Results

All participants had accessed HIV counselling and testing at least once at either a public primary health care facility or a non-governmental mobile service between February and April 2011.

### Demographics

The demographic characteristics of the participants who attended mobile and clinic HCT were similar (Table 2). Twelve females and 4 males with an age range of 19–60 years (median age 28 years) were interviewed; 8 each per HCT provider type. Males were under represented in both groups, with 3 of the 4 male participants having accessed mobile HCT. All participants could speak and understand English, but most preferred to answer the questions in their first language, in order to better express themselves. Overall, 10 participants were black and spoke isiXhosa, while 6 were mixed race, of which 5 spoke Afrikaans and 1 spoke English during the interviews. Eleven participants reported that they had never been married and most participants had at least one child. Half had completed grade 12 schooling and 10 were not employed. Half of the participants reported to live in an informal structure (wood or tin shack).

**Table 2:** Demographic data of participants interviewed across mobile and clinic HCT

	Mobile HCT (*n* = 8)	Clinic HCT (*n* = 8)	Total (*n* = 16)
Ethnicity and sex			
Black males	2	0	2
Mixed race males	1	1	2
Black females	3	5	8
Mixed race females	2	2	4
Age groups			
18–25 years	5	1	6
26–30 years	1	2	3
31–50 years	2	4	6
>51 years	1	0	1
Marital status			
Married	2	3	5
Not married	7	4	11
Education			
Completed grade 12	6	2	8
Incomplete schooling	3	5	8
Employment			
Unemployed	5	5	10
Employed/other	0	1	1
Dwelling			
Formal (brick)	5	3	8
Informal (shack)	4	4	8

### Health seeking behaviour

Participants, irrespective of which service provider they visited, reported similar reasons for having an HIV test; availability of HCT, being affected by HIV and a perceived personal risk for HIV. They also reported similar reasons for choosing a particular service provider; accessibility, familiarity and acceptability of services. No differences in experiences or perspectives by age, sex or ethnicity were noted.

### Reason for seeking an HIV test

#### Opportunity

Participants who utilised mobile HCT reported that the opportunity to test presented itself because they walked past the pop-up tent offering the HCT service and would otherwise not have tested at that particular time: “On the way we saw the tents on the side of the road with a sign that says HCT.” (Female, 19 years). Opportunity was also created when staff actively mobilised community members to test: “I was busy in my garden… they asked if I would like to come for a TB test…when I got to the tent they told me about the HCT service and offered it to me.” (Male, 60 years). Opportunity to test was also realised when individuals accompanied a friend or family member who had come for an HIV test: “I accompanied my sister…she didn’t want to go alone” (Female, 38 years).

In the PHC facilities, HCT was also made available through the opportunity to test when someone visited the facility for another health reason. This was highlighted where HIV testing can be provided as part of an integrated clinical service, as one participant explained: “I got tested when I went for my [family planning] injection.” (Female 37 years).

#### Affected by HIV

Being personally affected by HIV through knowing someone who has HIV or who died of a related illness emerged as a common theme. Participants from both the public PHC facilities and NGO mobile services explained how they were either directly motivated by family and friends, to test for HIV: “I have a friend who is HIV, she would tell me to go and test…” (Female, 25 years) or indirectly, through illness of those close to them: “At home there is someone who is infected, so I just wanted to know mine [HIV status].” (Female 19 years). Loss of friends and family, through death was described as providing a strong motivation to seek out HIV testing: “I have a cousin who is HIV positive…she died in January…that was one of the things that motivated me a lot” (Female 21 years).

#### Perceived personal risk for HIV

Participants, irrespective of which service provider they utilized, were motivated to seek an HIV test if they believed that they were at risk, either because they believed their partner had been unfaithful and or because they had practised unsafe sex. Some female participants reported that they distrusted their partner, as one participant explained: “At the time I tested, I had a boyfriend whom I did not trust, and then I thought for my sake I needed to know my status.” (Female, 22 years). Another explained how suspicion of infidelity, combined with practicing unsafe sex was the motivating factor: “My boyfriend doesn’t want to use a condom…I also saw him with another woman…I was in fear not knowing how long it has been going on.” (Female, 17 years). In some cases, the presence of multiple partners was the motivator: “I found out he was having an affair and decided to go for a test.” (Female, 25 years). One male participant reported that his multiple partnerships motivated him to test for HIV: “I have these girls…sometimes I don’t use condoms.” (Male, 22 years). One female indicated that recurring sexually transmitted infections motivated her to test: “I went for a test after I kept getting [sexually transmitted] infections.” (Female, 39 years).

Not everyone reported an underlying reason for having a test. Some reported that they wanted to know their HIV status. A participant explained: “I was just curious to know my status.” (Female, 30 years).

### Reason for choice of service provider

#### Accessibility

Participants highlighted accessibility of the service provider as a factor when choosing either a NGO mobile or public PHC facility. They reported that both service providers were accessible because they were within walking distance and mostly involved no transport costs. For NGO mobile services, access was often on the spur of the moment. A taxi driver used the mobile HCT service during a break in his work schedule: “I was parked at the taxi terminus waiting for my taxi to fill up. I could just walk over to them [the tents]), it was very convenient to me.” (Male, 38 years). Two participants who utilised public PHC facilities reported that HCT was accessible because it was offered as part of other health services: “[HIV] testing is provided automatically with family planning.” (Female, 36 years).

#### Familiarity and acceptability

Previous experience and satisfaction with the service received also played a role in the choice of provider. Respondents who utilised NGO mobile services reported: “I always go to the tents…people say you wait very long at the clinic.” (Female, 38 years). Participants who utilised public PHC facilities reported: “I knew the [HCT] service was rendered there [at the facility].” (Female, 32 years). One participant indicated that lack of familiarity with tents dissuaded her from accessing them: “I don’t know these other places like these tents…” (Female, 21 years). For another participant, the specialised and targeted service provided by the NGO mobile services had advantages. He explained: “Tents are different from clinics…in clinics you must wait with sick people.” (Male, 22 years).

### Experiences of HCT

Participants reported predominantly positive experiences irrespective of where they accessed HCT, though there were some complaints about HCT services within public health facilities. Concerns about stigma were reported for both services. The shorter waiting times at NGO mobile services distinguished it from the public PHC facilities.

#### Waiting time

The short waiting time at mobile HCT was highlighted as a major positive factor: “I sat outside for 2 min and then they called me in.” (Female, 22 years). Three participants with experience of attending public PHC facilities and NGO mobile services compared the waiting time at the mobile service to the longer waiting times experienced at the public facility: one of them said: “The clinic has long queues and you wait forever…at the tents there are no queues.” (Female, 24 years).

Long waiting times was a common experience for those participants who accessed HCT services at public PHC facilities. One participant said: “You wait first…I waited about 2 h.” (Female, 30 years). By contrast, one participant reported no waiting time when accessing HCT at a public PHC facility: “I just went in.” (Male, 23 years).

#### Staff attitude, competence and trust

Staff attitude, competence and perceived trustworthiness emerged as factors influencing the HCT experience. The friendliness of staff made a particular impression on participants. Several made explicit reference to this, especially at the NGO mobile sites: “People are nice and friendly…I was welcomed with a big smile.” (Female, 19 years). A few participants compared their experience of staff at the mobile service as being more favourable than at the public facilities: “People are friendlier…unlike in the clinic…staff at the clinic are tired and don’t feel like talking to you.” (Female, 22 years). Participants who attended public PHC facilities, more often reported a mixed picture: “Clinic staff are friendly, but there are a few nurses that are rude.” (Female, 30 year).

Overall participants reported positive perceptions around staff proficiency. Respondents who visited the NGO mobile service were generally positive: “Counsellors are good. They know how to tell people their results” (Male, 38 years) and “I felt the nurse pressing against me [when doing the finger prick] and she said she was finished; it was not painful.” (Female, 27 years). Similarly, participants who attended a public PHC facility reported being pleased with the level of competence: “The [nursing] sister explained what she was going to do.” (Female, 39 years).

All participants reported a level of trust in the staff, irrespective of where they accessed HCT. A respondent who visited the NGO mobile service reported: “I trusted them with my information.” (Female, 24 years) and one who attended a public PHC facility said: “I sat privately… everything was very confidential… I felt I could trust them.” (Female, 39 years).”

#### HCT setting

Participants described the setting in terms of cleanliness, privacy and perceived stigma. Cleanliness of the surroundings in both the public PHC facilities and NGO mobile services contributed to a sense of confidence in the services. Two participants were particularly impressed by the NGO mobile service, commenting that: “The place was neat and clean, it looked like a real health facility.” (Male, 60 years).

Participants experienced the NGO mobile service as private: “there was privacy and no one will listen to what you are saying” (Female, 27 years). Participants had differing views on privacy within the public PHC facilities, with some noting it lacked privacy, whilst others found it offered sufficient privacy. Privacy in the consultation room was sometimes affected by other staff or patients entering the room or because entering or exiting the counselling room indicated that you were there for an HIV test. One participant summed up a common sentiment: “while in the [counselling] room, people just knock and come in, so I feel the tents are more private and confidential.” (Female, 22 years). In contrast, another participant who had also attended a public PHC facility had a more private experience of HIV testing: “Everything was private…no one will even knock on the door.” (Female, 39 years).

Participants commented on stigma being a concern at both public PHC facilities and NGO mobile services. One participant was concerned about stigma at the health facility and compared the mobile service more favourably: “At the clinic most people know you, the minute you enter the room they know what you are there for and decide that you are HIV positive....There is no discrimination [at the tents]…all my worries about getting tested were put at ease.” (Female, 22 years). Another participant expressed the opposite sentiment: “At the clinic at least I am ok because there is a lot happening there, but at this one [tents] they know straight away there is nothing else but [HIV] testing here.” (Female, 21 years).

## Discussion

This study compared client health seeking behaviour and experiences at two types of HCT services; those provided by non-governmental organisations that provided mobile services and public primary health care facilities, offering HCT services from fixed sites.

Irrespective of where participants obtained their HIV test, health seeking behaviour was related to the opportunity to test, being affected by HIV and a perceived personal risk for HIV. These findings are consistent with other studies in sub-Saharan Africa which showed that having an HIV test was associated with knowing the location of a test site [17, 18], personally knowing someone with HIV [17, 19, 20], and a perceived sense of being at risk for HIV infection [17, 18, 21, 22].

The concept of availability is concerned with an adequate supply of services [9] and the opportunity to obtain an HCT service by those who need it when they need it [23]. This study has highlighted that opportunity to obtain an HIV test does play a role in the utilization of HCT services. Providing an HCT opportunity, whether through a mobile service provided at a busy transport hub or an integrated health service at a public health facility, makes HCT physically available. Mobile services appear to have ‘opportunistic’ clients compared to health facilities, as they are able to attract those who are just walking past and who would otherwise not have tested for HIV at that particular time. Mobile HCT plays a role in increasing access for those who were not contemplating testing until this opportunity arose.

Opportunity to test is closely aligned with accessibility, which emerged as an important consideration for participants regardless of which service they attended. Participants who attended the NGO mobile services spoke about “just passing by”. The accessibility of mobile services may lie in the increased proximity of the testing site to the surrounding community; reducing travel time and cost for those who do not stay close to a clinic. This is in line with studies in sub-Saharan Africa which have shown that distance to testing site and travel cost are barriers to HIV testing [19, 24, 25]. Those who attended public PHC facilities reported that the facility was in close proximity to where they live. Health facilities are able to provide HIV testing through integrated health care, for example reproductive health services, making HIV testing very accessible to those who attend these services. For these clients, there is no additional travel time or cost to acquire an HIV test.

The convenience of having physical access to the opportunity to test for HIV played a role in participants’ decisions to test for HIV. The accessible opportunity that mobile HCT offers should be considered when aiming to increase access to HCT overall, as mobile HCT can provide services to those who are passing by and would not have accessed an HIV test at that particular time. Currently, mobile services are limited in the number of related health services they offer compared to public primary health care facilities. Future research could determine if a wider variety of health services at mobile HCT could attract a higher number of people.

Improving availability of an HCT service requires consideration around the manner in which the service is organised to meet the needs of the client. Within this study, waiting time emerged as a differentiating factor between the two service types. Longer waiting times were consistently reported as a source of dissatisfaction in health facilities and may be potential barriers to access. Many South African studies have reported client dissatisfaction with long waiting times with other health services [26–29]. Shorter waiting time encouraged the use of NGO mobile services and may therefore provide an avenue to reduce the time at HCT and thereby expand uptake. However, a higher utilisation of mobile services may put a strain on waiting times. It is also important to consider that shorter waiting times is not a unique feature of the NGO mobile service and could also be achieved within a public clinic setting by fast-tracking clients who only want to access HCT services.

Availability is just one dimension of access. Understanding client experiences is also important for developing strategies to strengthen health systems and improve access to services. Acceptability indicates a match between service provision and the expectation of the user [23]. Although acceptability is critical to ensuring that an individual uses a service [14], it is a subjective concept, heavily dependent on the user’s expectations. Although this study did not explicitly measure satisfaction, the reported experiences indicate levels of satisfaction, which determine future utilisation of health care services and are ultimately linked to access [27].

Participants reported overall positive experiences irrespective of where they accessed services. Staff attitude, competence and trust emerged as factors influencing acceptability. Participants who accessed mobile services experienced friendly competent staff that they felt they could trust with keeping their HIV results confidential. Reports regarding the friendliness of health facility staff were contradictory and instances of poor staff demeanour were noted. While some South African studies reveal high levels of satisfaction with public health care providers [26, 30], others have reported that staff did not treat patients with sufficient respect [27, 31]. Anticipated disrespectful treatment has been shown to be partly accountable for delayed care seeking [13]. Conduct of nurses is a core element by which clients judge health services [32]. Although this study did not identify reasons for poor staff demeanour, shortage of staff and management, inadequate resources, high workloads and stress and burnout have been identified as major challenges faced by nurses [33, 34] and may have played a role in the cases where participants reported incidents of poor staff demeanour.

Healthier working environments, with adequate resources and reduced stress have been linked to positive client experiences [35]. Interventions that strengthen management capacity [36], ensure teamwork among healthcare providers [37] and enhance trust between health care providers and patients [38] are required for a healthier working environment. Within the NGO mobile HCT services, professional nurses are employed in a management capacity and receive continual training in performance management skills. This includes developing skills in data evaluation, which allows the professional nurses to better understand their HCT data, identify gaps in the services and generate and implement plans that address these gaps. In this manner professional nurses are able to provide strategic direction to their teams. General staff wellness is addressed through regular debriefing sessions. These sessions are hosted bi-monthly by psychologists and social workers and are attended by the entire mobile HCT team. The sessions provide psychosocial support and in addition aim to develop communication and teamwork. These interventions may have played a role in the friendly and competent service reported by participants who accessed mobile HCT.

Participants did not have concerns about the cleanliness of the HCT setting, but were concerned about the risk of being stigmatized in both the health facility and mobile settings. This aligns with previous work in high HIV prevalence settings that showed that concerns about stigma did not differ between HCT services in integrated or non-integrated health facility settings [39]. In health facilities, perceived stigma was associated with overcrowding and lack of private spaces, whilst at the mobile service; it was due to the public placement of the tents. Further investigation into the experience of stigma and ways to reduce stigma is required to help inform health authorities on ways to limit real or perceived stigma associated with HCT.

This study compared two HCT service providers across two dimensions of access; availability and acceptability. Both dimensions are important considerations when aiming to increase access to HCT services at these health service providers. Future operational research could explore the dimensions of access further within the South African context. Research is required to assess the feasibility of government outreach services, and whether these can increase availability of services, by accessing different populations to those presenting at health facilities. Future studies are also needed to determine the impact of interventions designed to produce a healthy working environment. Randomized control trials could be utilised to determine if such interventions impact on the acceptability of health services within this context. Affordability is a third dimension along which access is measured and should also be taken into consideration in future studies.

### Strengths and limitations

The study was conducted within a limited geographical area, which limited the number of PHC facilities and mobile services included. The sample of participants was relatively small. However, the findings align with those in large representative sample surveys [31] and provide significant insight into individual perspectives and circumstances motivating HCT utilisation and provider choice.

The translation process was not checked for quality and no back translation was done, which may have limited the quality and depth of the English transcription - some nuances in the language use of patients may not have been sufficiently captured. Not translating the interview guide may have limited the consistency of the translation of questions between interviewers, but all interviewers spoke and understood English well.

The length of the interviews varied, with half of the interviews taking 20 to 30 min, and half 30 to 60 min. This is directly attributed to the amount of probing and may have limited the depth and quality of the data collected. Whilst all interviews addressed the main guiding questions, depth and quality of data was potentially limited in shorter interviews.

The study only takes into consideration the health seeking behaviour of those who had an HIV test. In order to better understand barriers to accessing HCT, future studies should include participants who have never tested.

The study does provide a unique comparative analysis of NGO mobile services and public sector primary health care facilities providing HCT services within a specific geographical area that is representative of densely populated, low socio-economic urban settings, where the highest levels of HIV infection are found. It also highlights some of the key issues that affect utilisation of HCT services from a client perspective in such a setting, which provides insights to guide policy makers and other stakeholders in exploring strategies that can bring us closer to the goal of universal HCT coverage.

## Conclusion

Realised access to HCT is dependent on a number of factors, some of which have been considered within the dimensions of availability and acceptability of HCT services, as reported by those who utilised either a NGO mobile service or a public PHC facility. Both public sector and mobile NGO HCT services were perceived as available and acceptable for most. The latter provided an accessible opportunity for those who would otherwise not have tested at that time. Policy makers should consider diverse HCT strategies that take into account availability and acceptability of services. In doing so they should consider the perceptions and experiences of those who have accessed HCT services when increasing access to HCT.
